# Succession and assembly mechanisms of seawater prokaryotic communities along an extremely wide salinity gradient

**DOI:** 10.1111/1758-2229.13188

**Published:** 2023-08-03

**Authors:** Xiaoyan Guan, Zelong Zhao, Jingwei Jiang, Lei Fu, Jiaojiao Liu, Yongjia Pan, Shan Gao, Bai Wang, Zhong Chen, Xuda Wang, Hongjuan Sun, Bing Jiang, Ying Dong, Zunchun Zhou

**Affiliations:** ^1^ Liaoning Key Laboratory of Marine Fishery Molecular Biology, Liaoning Key Lab of Germplasm Improvement and Fine Seed Breeding of Marine Aquatic Animals Liaoning Ocean and Fisheries Science Research Institute Dalian Liaoning People's Republic of China; ^2^ Dalian Salt Chemical Group Co., Ltd Dalian Liaoning People's Republic of China

## Abstract

Salinity is an important environmental factor in microbial ecology for affecting the microbial communities in diverse environments. Understanding the salinity adaptation mechanisms of a microbial community is a significant issue, while most previous studies only covered a narrow salinity range. Here, variations in seawater prokaryotic communities during the whole salt drying progression (salinity from 3% to 25%) were investigated. According to high‐throughput sequencing results, the diversity, composition, and function of seawater prokaryotic communities varied significantly along the salinity gradient, expressing as decreased diversity, enrichment of some halophilic archaea, and powerful nitrate reduction in samples with high salt concentrations. More importantly, a sudden and dramatic alteration of prokaryotic communities was observed when salinity reached 16%, which was recognized as the change point. Combined with the results of network analysis, we found the increasing of complexity but decreasing of stability in prokaryotic communities when salinity exceeded the change point. Moreover, prokaryotic communities became more deterministic when salinity exceeded the change point due to the niche adaptation of halophilic species. Our study showed that substantial variations in seawater prokaryotic communities along an extremely wide salinity gradient, and also explored the underlying mechanisms regulating these changes.

## Graphical abstract

Succession and assembly mechanisms of prokaryotic communities along an extremely wide salinity gradient.

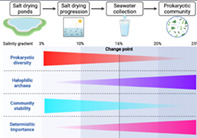

## INTRODUCTION

Salinity has been considered as an interesting environmental filter for advancing microbial ecology (Sultanpuram & Mothe, 2019). The most representative habitat is the marine environment, which has different microbial communities from freshwater environments (Sun et al., 2020). However, the salinity of oceans is only about 3%, there are many other ecosystems with higher salt concentrations on the Earth. For example, there are salt lakes with extremely high salinity, which occupy a unique niche (Kumar et al., 2020). In addition, salinization of soils induced by agriculture and high salt wastewater produced by industries have been considered as one of the most pressing environmental challenges for several decades (Rath & Rousk, 2015; Shi et al., 2020). Moreover, high salinity seawater in the salt drying process can also be used to breed artemia or high‐quality shrimps (Van Stappen et al., 2020). In these extremely salty environments, microbial communities play key roles in ecological functioning (Oren, 2015). In general, the concentration of salt in the environment has a negative effect on microorganisms by inhibiting respiration and growth (Setia et al., 2011). However, some halophilic species can be enriched in high salt environments through physiological adaptations (Cui & Dyall‐Smith, 2021), and such species would be able to counteract some of the negative effects of salinity through interspecies interactions (Jones et al., 2018). Importantly, microbial community in the salt drying ponds is inextricably linked to the quality of salt production (Wei et al., 2022). It should be noted that there is still a debate over the extent to which the changes in salinity levels control microbial community divergence (Hollister et al., 2010; Yang et al., 2016). Salt drying process covers a wide salinity gradient from the normal seawater to supersaturated brine. Thus, it is significant to understand the adaptation mechanisms of microbial communities in such an extensive range of salinity gradients during salt drying process, which could not only explore the microbial community evolution under extreme salinity but also improve the quality of salt or other related products.

Nowadays, questions behind the changing microbial communities because of salinity are more focused on the consequences and mechanisms of community changes. In diverse environments, network analysis has been used to investigate microbial community co‐occurrence patterns (Fan et al., 2018; Liu et al., 2022; Zhou et al., 2021). In a co‐occurrence network, species are represented by nodes and interactions between species are represented by links, which can characterize the complexity of microbial communities (Hirano & Takemoto, 2019). In general, more complex microbial communities could possess high function redundancy due to the overlapping metabolic activities of different members (Moya & Ferrer, 2016), thereby deciphering more powerful environmental adaptation (Tian et al., 2022). Besides, a microbial community that tends to exhibit relative stability could be more resistant to the external disturbances (Coyte et al., 2015), which can also be characterized by some features of co‐occurrence networks (Yuan et al., 2021). In the past, a number of studies have examined the relationship between network complexity and stability, but the results remain controversial (Landi et al., 2018; Toju et al., 2017). Although it is well documented that changes in salinity affect microbial communities (Mo et al., 2021), variations in co‐occurrence network of microbial communities' response to extreme salinity stresses has not been investigated yet.

Microbial ecology seeks to understand how ecological processes shape the assembly of microbial communities (Martiny et al., 2006). Network analysis can reveal the consequences of microbial community changes, but the underlying mechanisms behind these changes remain unclear. In accordance with the microbial ecological theories, the balance of two ecological processes, namely deterministic and stochastic, shapes the assembly of microbial communities (Zhao et al., 2022). When communities are governed by deterministic processes, the presence of species can be predictable because species occupy specific ecological niches (Vanwonterghem et al., 2014). In contrast, it is suggested by neutral‐based theory that multiple species can coexist in similar or overlapping habitats and the relative abundance of species changes according to stochastic fluctuations (Sloan et al., 2006). According to time, space, and ecosystem, the relative importance of stochastic and deterministic processes in the microbial community assembly is variable (Evans et al., 2017). Over the past decade, several studies have characterized the effect of salinity on the shift of aquatic or soil microbial communities in some natural or artificial environments (Hou et al., 2017; Pinnell & Turner, 2020; Zhang et al., 2019; Zhang, Bai, et al., 2021). However, a relatively small amount of information is available about the changes of microbial communities and their underlying ecological processes along an extremely wide salinity gradient due to the narrow range of salinity in any single dataset or ecosystem. Salt drying system is an artificial pond, in which seawater gradually dehydrate to crystallize salt through the action of sunlight. In a salt drying factory, there will be a large number of independent salt drying ponds. Due to the different injection times of seawater, there will be salt drying ponds with different salinity at the same time point. Bringing together a group of salt drying systems with different salinities is ideal for disentangling these open questions.

In this study, some currently unsolved concerns were addressed, which are: (i) how extreme salinity stress affects the diversity, composition, and function of seawater prokaryotic communities? (ii) how network complexity and stability of prokaryotic communities vary under extreme salinity gradient? and (iii) which of the stochastic and deterministic processes dominates the seawater prokaryotic community assembly along the extreme salinity gradient? To achieve these objectives, we tracked the dynamics of seawater prokaryotic communities along the extreme salinity gradient in salt drying tanks by high‐throughput sequencing of the 16S rRNA gene. Based on the sequencing dataset, variations in diversity, composition, and function of seawater prokaryotic communities were analysed first, and a potential change point of salinity was observed. Subsequently, the complexity and stability of prokaryotic co‐occurrence networks between both sides of the salinity change point were compared. Moreover, the assembly mechanisms of prokaryotic communities along the extreme salinity gradient were determined by the infer‐community assembly mechanisms by phylogenetic‐bin‐based null model analysis (iCAMP). The findings of this study can greatly expand our understanding of the ecological adaptation and succession mechanism of seawater prokaryotic communities under extremely high salt environments.

## EXPERIMENTAL PROCEDURES

### Sample collection and DNA extraction

Seawater samples were collected in a series of salt drying tanks from the Dalian Yan Hua Group Co., Ltd (Figure S1). Water salinity was measured by a water quality analyser, and tanks with a salinity gradient from 3% to 25% were collected. Each of six salt drying tanks with salinity at ~3%, 6%, 9%, 11%, 13%, 15%, 17%, 19%, 21%, 23%, and 25% were selected to collect seawater samples. A total of 66 seawater samples were collected in July 2022. For each sample, approximately 1 L of surface water was collected with a water sampler, temporarily held on ice, and immediately transport to laboratory. Each seawater sample was filtered via 0.22‐μm pore size polycarbonate membranes (142 mm diameter, Millipore, USA) using a peristaltic pump to concentrate microbes for DNA extraction. Total DNA was extracted from the filters using the PowerWater DNA isolation kit (QIAGEN, CA, USA) according to the manufacturer's instructions. The extracted DNA was examined by agarose gel electrophoresis (1% concentration) and NanoDrop ND‐1000 Spectrophotometer (NanoDrop, USA) was used to evaluate their qualities. All successfully extracted DNA was stored at −20°C until further application.

### High‐throughput sequencing and sequence data processing

The primers, 341F and 806R, were applied to amplify the V3‐V4 regions of prokaryotic 16S rRNA gene from each extracted DNA sample (Berg et al., 2012). The processes of PCR and sequencing library construction were consisting to our previous study (Zhao et al., 2022). These libraries were sequenced at BIOZERON Biotech. Co., Ltd in Shanghai, China, using an Illumina Novaseq6000 platform with a 250 bp paired‐end strategy. The sequenced paired‐end reads were appointed to the samples based on their unique barcode at the end of reverse primer. Then, low quality reads (average Phred scores <20, contained ambiguous bases, homopolymer runs >8, had mismatches in the primers, or sequence length <250 bp) were removed (Bokulich et al., 2013). Paired reads were assembled, chimeras were eliminated, and clean data were clustered into the amplicon sequence variants (ASVs) using the DADA2 plugin unit in the QIIME2 program (Bokulich et al., 2018). Singletons (the number of a specific ASV was one) were abandoned and all remaining ASVs were annotated to a taxonomy using the SILVA database (Release 138) (Yilmaz et al., 2014). The ASV abundance tables were normalized according to the sample with the lowest read number (33,295). Finally, the ecological function of prokaryotic communities was predicted the with the FAPROTAX software (Louca et al., 2016).

### Statistical analysis

All statistical analyses were implemented by R v4.1.0. Rarefaction and species cumulative curves of the sequenced datasets were extrapolated to assess the efficacy of sequencing depth and sample size (‘iNEXT’ package). Four alpha diversity indices of prokaryotic communities, related to different facets, including richness (Chao1), evolution (Faith's phylogenetic diversity, Faith_pd), diversity (Shannon), and evenness (Pielou's evenness index, Pielou_J), were calculated (‘vegan’ package). Variations in alpha diversity indices as well as the relative abundances of dominant functional terms and prokaryotes of seawater prokaryotic communities with different salinity were tested by Tukey's honest significant difference (HSD) test (‘multcomp’ package). In addition, the effects of salinity on the composition and function of seawater prokaryotic communities were assessed by principal coordinate analysis (PCoA) and the adonis test based on the Bray–Curtis distance (‘vegan’ and ‘ape’ packages). Furthermore, the niche breadth of dominant prokaryotic phyla along the salinity gradient were calculated to evaluate their environmental adaptation (‘TITAN2’ package).

A SEGMENTED method was performed to estimate the change point of salinity inducing the abrupt change of seawater prokaryotic communities (‘segmented’ package). Based on the estimated change point of salinity, a co‐occurrence network of seawater prokaryotic communities in samples lower or higher than the change point was constructed. Spearman's rank correlations among ASVs with a detected rate higher than 60% were calculated. The |correlation coefficient| was >0.6 and the *p*‐value was <0.05 was the thresholds for correlation between two ASVs. The modularity and topological parameters of networks were calculated and compared (‘igraph’ package). Robustness, vulnerability, and cohesion for co‐occurrence networks were calculated to evaluate the stability of seawater prokaryotic communities (Yuan et al., 2021). iCAMP was used to quantitate the assembly of seawater prokaryotic communities in response to salinity variations (Ning et al., 2020). The effect of salinity change on ecological processes was assessed using the standardized effect size (Cohen's d). The detailed statistical analyses for iCAMP results were performed according to the previous study (Wang et al., 2021).

## RESULTS

### Variations in the diversity of seawater prokaryotic communities along the salinity gradient

Prokaryotic communities of seawater in salt drying tanks with a salinity gradient were measured, and a total of 1,246,075 high‐quality reads were obtained (Table S1). These reads were clustered into 2802 ASVs, which were annotated into 34 phyla, 49 classes, 106 orders, 228 families, 565 genera, and 667 species. Almost all ASVs were annotated at the phylum level and more than 90% of ASVs were assigned to a genus. In contrast, only 68.93% ASVs were successfully annotated to a species (Figure S2). In addition, rarefaction curves based on the detected ASV numbers of each sample were all near the horizontal position (Figure S3), suggesting that the amounts of sequencing depth were enough to reflect the intact prokaryotic communities. Moreover, the species accumulation curves showed a continuous upward trend with the increase of sample size (Figure S4), suggesting that there might be some undetected rare species in seawater with high salinity.

**FIGURE 1:**
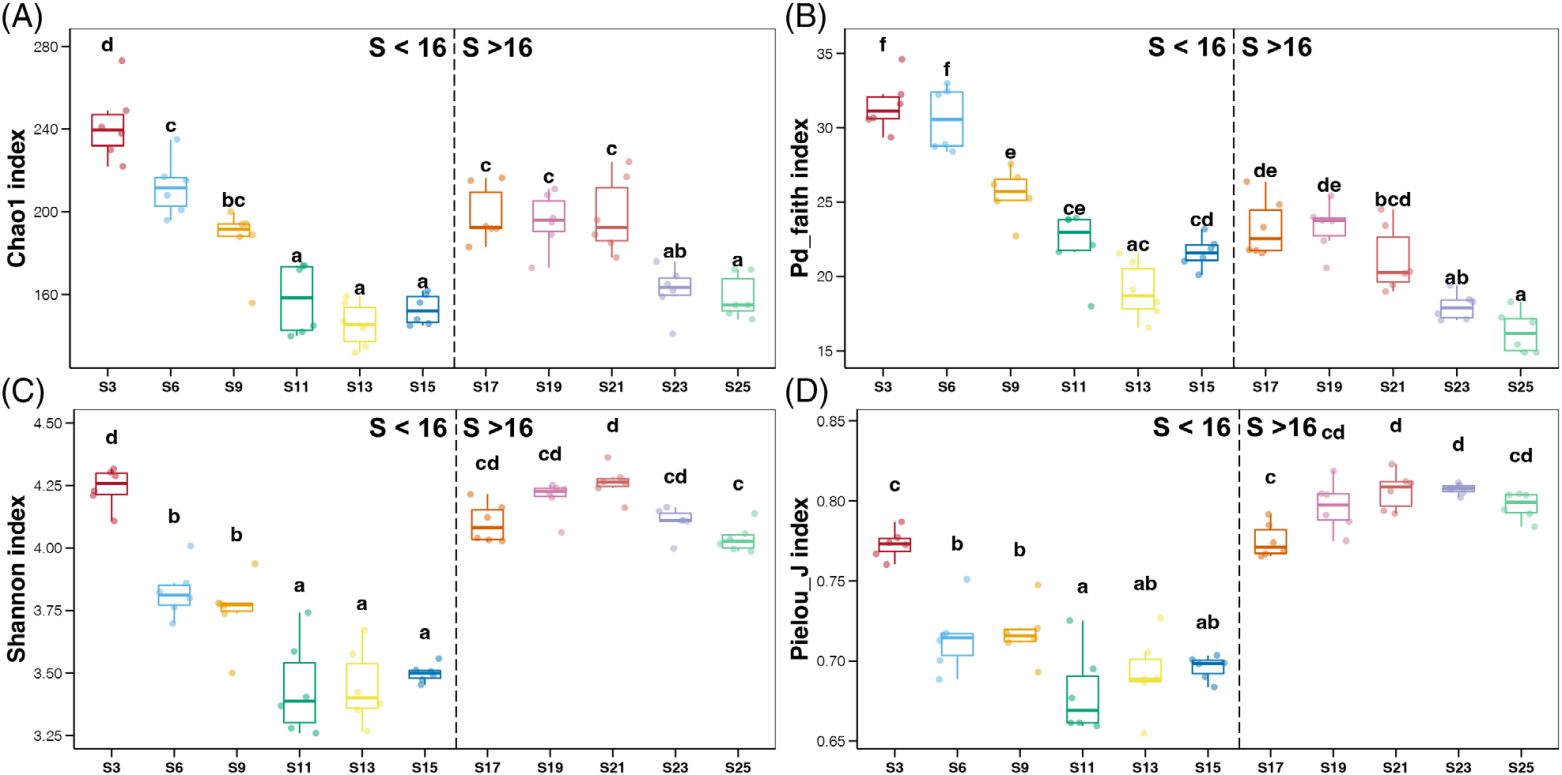
Variations in alpha diversity indices of seawater prokaryotic communities of salt drying tanks along the extremely wide salinity gradient. (A) Chao1; (B) Pd_faith; (C) Shannon; and (D) Pielou_J. Different lowercases letters above each box in the same sub‐Fig. represent significant differences between samples from different salinities (Tukey's HSD test, *p* < 0.05).

Variations in alpha diversity of seawater prokaryotic communities along the salinity gradient are shown in Figure 1. According to the change trend, we could observe a change point of salinity (~16%). When salinity was lower than 16, all four alpha diversity indices of seawater prokaryotic communities gradually decreased with an increase in salinity. When salinity reached 16, all four alpha diversity indices showed a sudden increase. Subsequently, the Chao1 and Pd_faith indices gradually decreased again with the increase in salinity (Figure 1A,B), but the Shannon and Pielou_J indices remained relatively stable (Figure 1C,D). These results indicated that extreme high salt conditions significantly impact the diversity of prokaryotes, and a potential threshold of salinity (~16%) on prokaryotic diversity was observed.

### Variations in the composition of seawater prokaryotic communities along the salinity gradient

**FIGURE 2:**
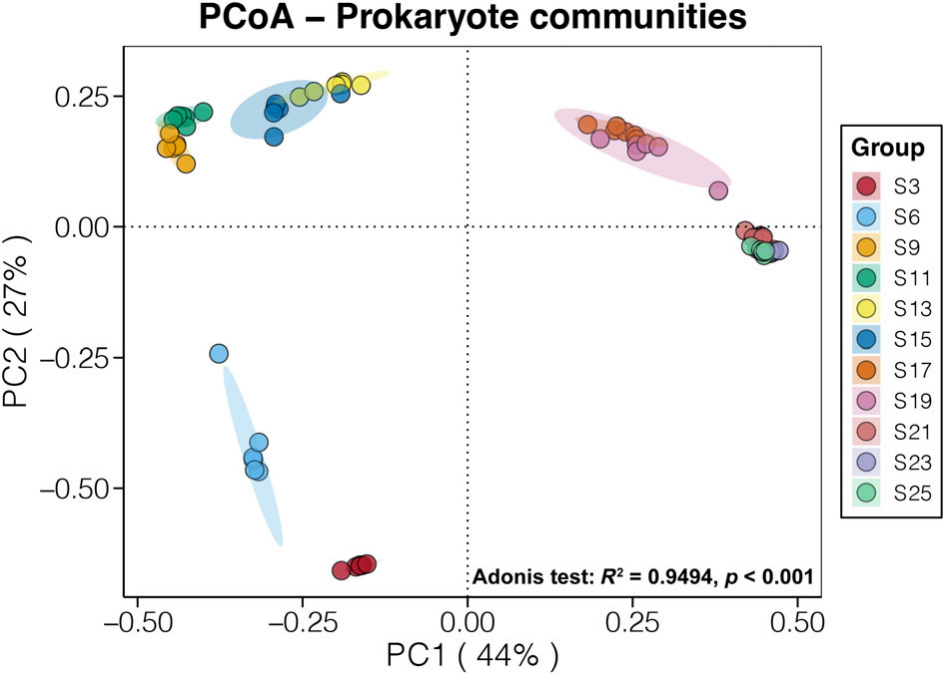
Principal coordinate analysis (PCoA) and the adonis test of seawater prokaryotic communities along the salinity gradient based on the Bray–Curtis distance.

PCoA based on the Bray–Curtis distance showed that seawater prokaryotic communities under different salinity were separately clustered (Figure 2). Samples under salinity lower and higher than 16% were separated by the PC1 axis, which explained 44% of the total community variations. In addition, the PC2 axis separated samples at low salinity (3% and 6%) with others, which explained 27% of the total community variations. The adonis test also confirmed the significant effects of salinity on seawater prokaryotic communities, which could explain 94.94% of the community variations. These findings confirmed the significant influence of salinity on the composition structure of seawater prokaryotic communities.

**FIGURE 3:**
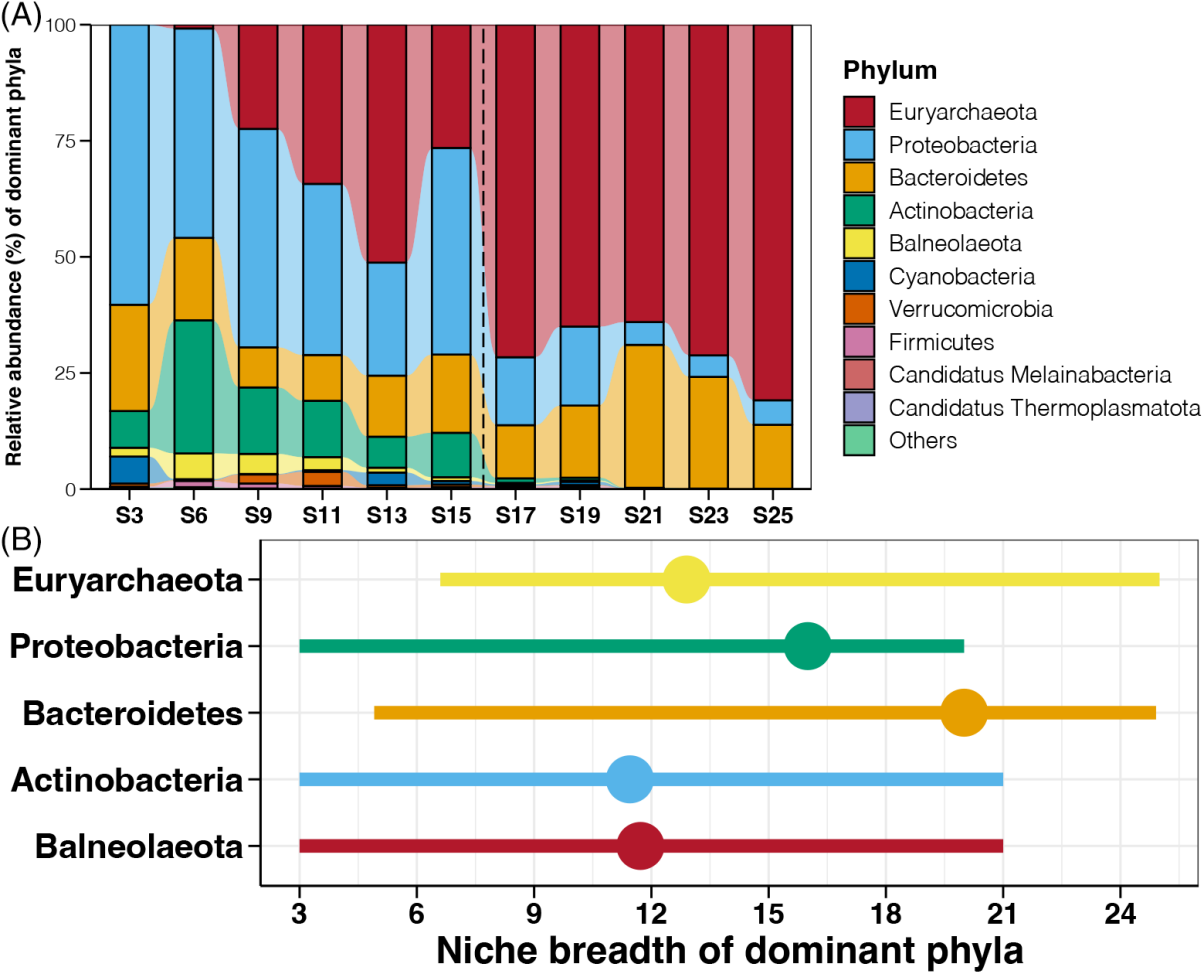
(A) Variations in relative abundances of the top10 prokaryotic phyla in seawater along the salinity gradient. (B) Niche breadth and change point of dominant prokaryotic phyla related to salinity variation.

Euryarchaeota (44.37%) was the most dominant prokaryotic phylum in the studied samples, followed by Proteobacteria (27.68%), Bacteroidetes (16.79%) and Actinobacteria (7.36%) (Figure S5). With the increase of salinity, the relative abundance of Euryarchaeota was gradually enhanced, but that of Proteobacteria continuously declined (Figure 3A and S5). In addition, some bacterial phyla, including Actinobacteria, Balneolaeota, Cyanobacteria, and Verrucomicrobia, were almost vanished in samples with salinity higher than 16% (Figures 3A and S6). The niche breadth of dominant prokaryotic phyla was also measured to assess the salinity adaptation of different species (Figure 3B). Euryarchaeota and Bacteroidetes could survive at extremely high salinities, but Proteobacteria, Actinobacteria, and Balneolaeota could not adapt to salinities higher than 21%. The point in Figure 3B represents the change point of different phyla, meaning that prokaryotes could be more abundant or wiped out when salinity higher than them. The change point for Euryarchaeota, Actinobacteria, and Balneolaeota was about 12%, while that for Proteobacteria and Bacteroidetes was about 16% and 20%, respectively (Figure 3B). At the genus level, some halotolerant genera, including *Halorubrum*, *Salinibacter*, *Haloquadratum*, and *Halobellus*, were enriched when salinity reached 13% (Figure S7), and were significantly more abundant in samples with salinities higher than 16% (Tukey's HSD test, *p* < 0.05, Figure S8). In contrast, several prokaryotic genera, including *Halogeometricum*, *Spiribacger*, *Halopelagius*, *Roseovarius*, and *Psychroflexus*, were more abundant in samples with salinities lower than 16% and almost disappeared when salinities reached 20% (Figures S7 and S8). Those results indicated that differences in salinity adaptation between different prokaryotes could be the reason for the variations in community compositions.

### Variations in function of seawater prokaryotic communities along the salinity gradient

**FIGURE 4:**
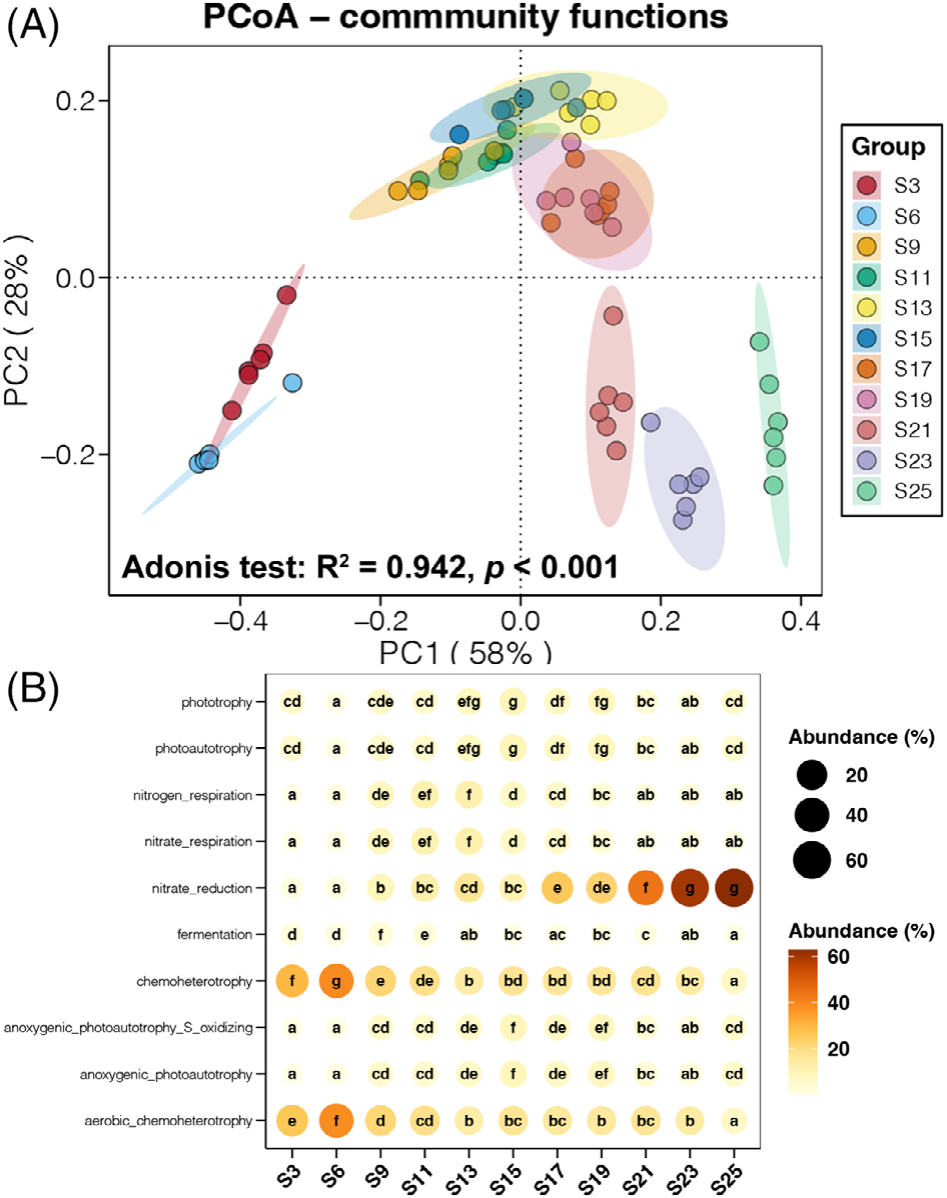
(A) Principal coordinate analysis (PCoA) and adonis test of the function structures of seawater prokaryotic communities along the salinity gradient based on the Bray–Curtis distance. (B) Bubble chart showing the relative abundances of functional terms in seawater prokaryotic communities along the salinity gradient. Different lowercase letters in each point at the same raw represent significant differences between samples with different salinities (Tukey's HSD test, *p* < 0.05).

Similar to the results of community composition, PCoA of function structure of seawater prokaryotic communities also clustered samples into three groups: S3 and S6, S9 to S19, and S12 to S25 (Figure 4A). Adonis test confirmed the significant influence of salinity on the function structure of seawater prokaryotic communities (*R* ^2^ = 0.942, *p* < 0.001). A more detailed estimation was attained for the variations in relative abundances of functional terms (Figure 4B). Generally, the relative abundances of nitrate reduction were gradually increased, but chemoheterotrophy decreased with the increase of salinity. In addition, functions related to photosynthesis and nitrogen respiration were more abundant in samples with salinity ranging from 9% to 19%. According to these results, salinity obviously affected the function of seawater prokaryotic communities.

### Co‐occurrence networks of seawater prokaryotic communities along the salinity gradient

**FIGURE 5:**
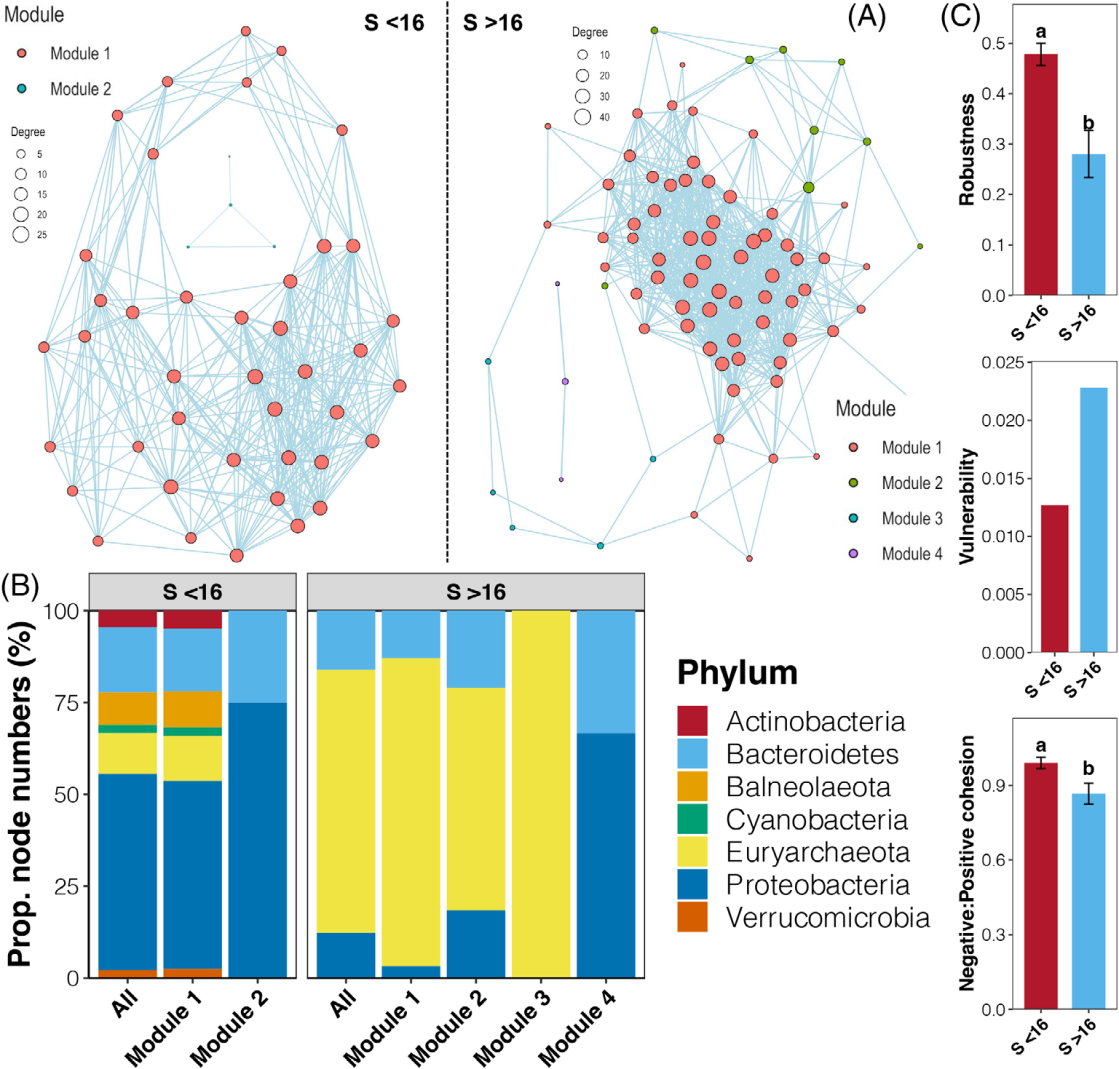
(A) Co‐occurrence networks of seawater prokaryotic communities with salinities lower or higher than 16%. Nodes belonging to different modules are labelled in different colours. (B) Composition of different modules at the phylum level. (C) Differences in the robustness, vulnerability, and negative: positive cohesion between networks of samples with salinities lower or higher than 16%. Different lowercase letters above each box in the same sub‐Fig. represent significant differences between samples from different groups (Tukey's HSD test, *p* < 0.05).

Based on the above analyses, we deduced that an obvious change point of salinity could exist, and the SEGMENTED method detected that the point where a sudden shift or change in seawater prokaryotic communities was at a salinity of ~16%. Based on these results, we assigned samples into two groups, including those with salinities higher than 16% (S > 16) and lower than 16% (S < 16). Co‐occurrence networks of seawater prokaryotic communities from these two groups were constructed respectively (Figure 5A), and the topological parameters of them are shown in Table S2. According to the topological parameters, the networks for both groups obeyed a power‐law distribution, suggesting a non‐random distribution pattern. In addition, the small world coefficient was higher than 1 for both networks, thus, indicating the small world characteristic. The networks of seawater prokaryotic communities in the S < 16 group had 45 nodes, 351 edges, and an average degree of 15.6. In contrast, the network for samples from the S > 16 group presented 81 nodes and 803 edges with an average degree of 19.827. These results revealed a more complex co‐occurrence pattern in seawater prokaryotic communities under higher salinity.

Based on the modularity analysis, two and four modules were identified from networks from the S < 16 and S > 16 groups, respectively. Proteobacteria dominated the modules from the S < 16 group with substantial members of Actinobacteria, Blaneolaeota, and Verrucomicrobia (Figure 5B). In contrast, ASVs belonged to Euryarchaeota occupied most of the modules from the S > 16 group (Figure 5B). More importantly, significantly higher robustness and negative: positive cohesion, but lower vulnerability, were found in the network of samples with salinities lower than 16 compared to those higher than 16 (Tukey's HSD test, *p* < 0.05, Figure 5C). Thus, such findings suggested a more stable network pattern of seawater prokaryotic communities under lower salinity.

### Assembly mechanism of seawater prokaryotic communities along the salinity gradient

**FIGURE 6:**
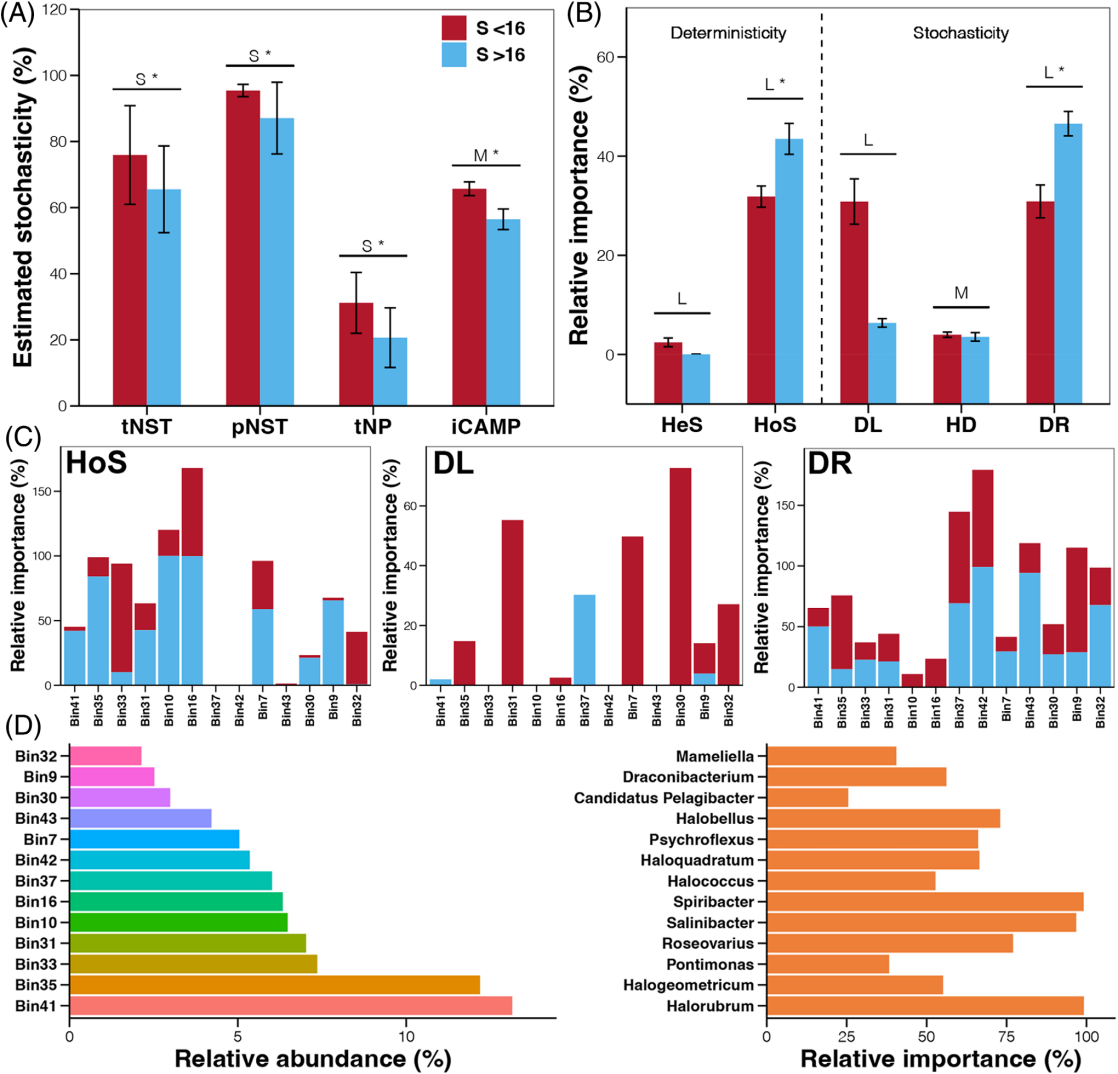
(A) Stochasticity estimated by different methods in samples with different salinities. (B) Relative importance of different ecological processes based on iCAMP between samples with different salinities. One‐sided significance based on the bootstrapping test was expressed as * (*p‐value* <0.05). L, M, S, and N represent large (|d| > 0.8), medium (0.5 < |d| ≤ 0.8), small (0.2 < |d| ≤ 0.5), and negligible (|d| ≤ 0.2) effect sizes of electrostimulation, based on Cohen's d (the mean difference between electrostimulation and control divided by pooled standard deviation). DL, dispersal limitation; DR, drift and others; HD, homogenizing dispersal; HeS, heterogeneous selection; HoS, homogeneous selection. (C) Relative importance of HoS, DL, and DR in the assembly of the major bins in the studied samples. (D) Relative abundance of major bins and the genus with greatest relative abundance in each bin.

First, the contribution of the ecological stochastic process was quantified by NP‐based neutral‐theory, NST based on taxonomic (tNST) or phylogenetic metrics (pNST), and iCAMP (Figure 6A). Although the specific values of different methods were unequal, all four methods revealed significantly lower ecological stochasticity in samples with salinities higher than 16% compared to those lower than 16%. Furthermore, the relative importance of different ecological processes, including heterogeneous selection (HeS), homogeneous selection (HoS), homogenizing dispersal (HD), dispersal limitation (DL), and drift (DR) was quantified based on iCAMP. Based on our results, HoS and DR were dominant ecological processes for the assembly of seawater prokaryotic communities along the extreme salinity gradient (Figure 6B). Moreover, both of them contributed more to the prokaryotic community assembly in samples with salinities higher than 16%, compared to those lower than 16% (Figure 6B). In addition, a remarkably higher contribution of DL was observed in samples from the S < 16 group (Figure 6B), which could be the reason for the higher stochasticity in those samples.

A useful aspect of iCMAP is that it can provide information on the relative importance of different ecological processes in different phylogenetic groups. According to the above results (Figure 6B), bins containing archaea and bacteria contributing to the salinity‐induced changes of HoS, DL, and DR were further analysed (Figure 6C). As the most abundant and high salinity (>16%)‐enriched genus, *Halorubrum* (about ~100% of total abundance in bin 41, Figure 6D) was governed by both HoS and DR (Figure 6C). For other high salinity‐enriched genera (Figures S7 and S8), *Salinibacter* (bin 10) was entirely governed by HoS, while *Haloquadratum* (bin 42) and *Halobellus* (bin 43) were entirely governed by DR (Figure 6C). In contrast, genera that were enriched in low salinity samples (<16%), including *Roseovarius* (bin 31) and *Psychroflexus* (bin 7), were entirely governed by DL (Figure 6C). Interestingly, according to our results, increases in the importance of the deterministic process for prokaryotic community assembly were not consistent with the salinity gradient, instead, it was a sudden and dramatic elevation when salinity reached the change point (~16%) (Figure S9). The relative importance of the deterministic process (HoS) for some dominant species clusters (bins 41, 35, 31, 10, 16, 7, 30, 9) were obviously higher in samples with salinities higher than the change point compared to those lower than the change point (Figure 6B,C). Moreover, no contributions of dominant stochastic process (DL) for some species clusters that almost disappeared in high salt samples, including bins 35, 31, and 7 (Figure S8), were detected (Figure 6B). Those results indicated that salt drying drove the assembly of seawater prokaryotic communities to be more deterministic through the selection of specific genera.

## DISCUSSION

There have been many studies on soil microbial communities responding to salinization (Haj‐Amor et al., 2022) and the influences of salinity on microbial communities in brackish aquatic environments (Zhang, Qi, et al., 2021). However, most of such studies only covered a narrow salinity range due to the limitations of geographic scales and environmental conditions. Therefore, knowledge gaps remain about the effects of salinity on microbial communities. The seawater salt drying process could be an ideal system to fill such knowledge gaps, as they can include an extremely wide salinity gradient from about 3% (normal seawater) up to complete salt saturation. Our study investigated variations in the prokaryotic communities in salt drying tanks over a wide salinity range covering the complete process of salt drying. This study observed significant variations in the diversity, composition, and function of prokaryotic communities during the salt drying process, and such research on the complete salt drying process will improve our understanding of the microbial ecology related to salinity.

Extreme salt environments provide a unique niche, which could be favourable to multiple specific microorganisms (Oren, 2015). Based on the results of diversity, composition, and functional analyses, a potential change point of salinity (~16%) was identified. When salinity exceeded the change point, the relative abundance of bacteria among prokaryotic communities was significantly decreased, while the abundance of archaea was enriched. Similar results have also been found in other studies of hypersaline environments (Guerrero, 2013). In samples with salinities exceeding the change point, we found some enriched genera belonged to the extremely halophilic archaea class, Halobacteria, which are mainly aerobic red‐pigmented chemoheterotrophs (Oren, 2019). Among them, strains belonging to the genera *Halorubrum*, *Haloquadratum*, and *Halobellus* presented as orange‐red or bright pink colonies, which could explain the polychrome appearance of salt drying tanks (McGenity & Grant, 2015). As photoheterotrophs, Halobacteria can eat organic compounds to provide themselves with materials to grow, but they also can use light energy to generate ATP (Oren, 2019). If Halobacteria are deprived of light, they will consume more organic matters to provide for their energetic needs (Yang et al., 2020). In addition, rhodopsin present in membrane of Halobacteria also act as the chloride ion pump to prevent the osmotic pressure of Halobacteria cells (Essen, 2002). Halobacteria serve as the primary food source for filter feeders such as brine shrimp and contribute largely to the food web in hypersaline environments (Grote & O'Malley, 2011). Therefore, salt drying systems require sufficient light to maintain the stability of its food web, which could improve the quality of shrimps breeding in them. Moreover, *Halorubrum* was the most dominant genus and can produce nitrite from nitrate (Yim et al., 2014), which is consistent with the level of nitrate reduction detected in this study. Thus, the nitrite content of sea salt should be considered due to its potential toxicity.

By examining the dynamics of prokaryotic communities during the salt drying process, our study provided several important insights into the roles of salinity in mediating the complexity and stability of seawater prokaryotic co‐occurrence networks. Prokaryotic co‐occurrence networks from salinity groups lower or higher than the change point was obviously different. Higher salinity often acts as a deterministic filtering factor to select specific microorganisms and results in a less complex community (Zhang, Bai, et al., 2021). However, our results revealed a more complex network structure in samples with higher salinities than the change point. More neutral taxa that are less responsive to salinity could prevail in prokaryotic communities with lower salinities (Burns et al., 2016). Although neutral taxa provide higher richness, the relationship between species is not close (Chave, 2004). In contrast, halophilic species living in extreme high salt conditions are more likely to coexist within the same community (Letten et al., 2017). The close interactions between many halophilic species may be the reason for more complex network patterns in samples with salinities higher than the change point.

Moreover, the relationship between the complexity and stability of microbial communities is still hotly debated with controversial findings (Landi et al., 2018). Some studies reported a high complexity of microbial community networks with high stability (Santolini & Barabási, 2018). In this study, our results revealed that network stability decreased as the complexity of seawater prokaryotic communities increased during the salt drying process. Cooperating networks of microbes can be efficient but often unstable, while the introduction of species to enhance competition can stabilize the cooperating network (Coyte et al., 2015). Under high salt conditions, microorganisms need to work closely to resist salinity pressure, while under low salt conditions, microorganisms with diverse functions will increase competition to improve community stability (Chase, 2011). Based on the above statements, species richness and functional traits should be considered when investigating the relationship between the complexity and stability of microbial communities.

In a ‘harshness’ environment, deterministic processes can increase their importance to microbial community assembly by influencing the fitness of the microbes, and thus, changing their composition and distribution. The results of assembly mechanism analyses in this study revealed that deterministic processes overwhelmed stochastic processes in determining the seawater prokaryotic communities in samples with salinities higher than the change point. Such findings were in agreement with other studies that focused on the microbial communities in aquatic environments with different salinities (Zhou et al., 2021). In general, high salinity, as a selective filtering factor, may select specific taxa to form a deterministic dominant microbial community (Zhang et al., 2019). As prokaryotes react rapidly to environmental perturbations, it is no surprise that environmental filtering was the most dominant process shaping the community of salt drying ecosystems. Moreover, we observed a sudden change in the assembly mechanism of prokaryotic communities when salinity exceeded the change point (~16%). Under this circumstance, some halophilic genera were rapidly enriched, while the relative abundances of other neutral taxa declined. When the salinity reached the change point, the environmental adaptation of dominant prokaryotic phyla varied significantly and differences in the niche breadth between such taxa could be one of the explanations for this phenomenon (Chen et al., 2021).

## CONCLUSIONS

In summary, this study provided the first molecular perspective for the effects of extremely wide salinity gradients on seawater prokaryotic communities collected during the complete salt drying process. The diversity, composition, and function of seawater prokaryotic communities varied significantly along the salinity gradient, with the enrichment of specific halophilic archaea. Based on the dynamics of seawater prokaryotic communities, a change point of salinity (~16%) was identified, which caused a sudden and dramatic variation in seawater prokaryotic communities. More importantly, our work revealed that prokaryotic co‐occurrence networks increased in complexity, but weakened in stability when salinity exceeded the change point. The relative importance of deterministic processes for the assembly of seawater prokaryotic communities was strengthened when salinity exceeded the change point. Our findings contribute valuable insights into the understanding the microbial ecology related to salinity.

## AUTHOR CONTRIBUTIONS

**Xiaoyan Guan:** Conceptualization (equal); funding acquisition (equal); project administration (equal); writing – original draft (equal). **Zelong Zhao:** Data curation (equal); formal analysis (equal); visualization (equal). **Jingwei Jiang:** Conceptualization (equal); writing – review and editing (equal). **Lei Fu:** Investigation (equal); resources (equal). **Jiaojiao Liu:** Investigation (equal); resources (equal). **Yongjia Pan:** Formal analysis (equal). **Shan Gao:** Methodology (equal). **Bai Wang:** Investigation (equal); validation (equal). **Zhong Chen:** Project administration (equal). **Xuda Wang:** Methodology (equal). **Hongjuan Sun:** Investigation (equal). **Bei Jiang:** Resources (equal). **Ying Dong:** Project administration (equal); supervision (equal). **Zhunchun Zhou:** Conceptualization (equal); funding acquisition (equal); project administration (equal); supervision (equal); writing – review and editing (equal).

## CONFLICT OF INTEREST STATEMENT

The authors declare that they have no known competing financial interests or personal relationships that could have appeared to influence the work reported in this paper.

## Supporting information


**Table S1.** Statistics of sequencing data.
**Table S2.** Topological parameters of co‐occurrence networks of prokaryote communities.
**Figure S1.** Pictures of sampling sites.
**Figure S2.** Statistics of taxonomy annotation.
**Figure S3.** Rarefaction curves.
**Figure S4.** Species accumulation curve.
**Figure S5.** Dominant prokaryotic phyla.
**Figure S6.** Differences in dominant prokaryotic phyla among samples with different salinity. Different lowercases letters above each box in the same sub‐figure represent significant differences among samples from different salinity (Tukey's HSD test, *p* < 0.05).
**Figure S7.** Relative abundances of dominant prokaryotic genera.
**Figure S8.** Differences in dominant prokaryotic genera among samples with different salinity. Different lowercases letters above each box in the same sub‐figure represent significant differences among samples from different salinity (Tukey's HSD test, *p* < 0.05).
**Figure S9.** Ratio of deterministic and stochastic processes to assembly of prokaryotic communities.

## Data Availability

All raw sequences of seawater prokaryotic communities studied in this study have been submitted to the NCBI Sequence Read Archive (SRA) database under the BioProject number PRJNA944819.
