# Supplementary material for: Succession and assembly mechanisms of seawater prokaryotic communities along an extremely wide salinity gradient
Source: Environ Microbiol Rep. 2023 Aug 3;15(6):545–56. doi: 10.1111/1758-2229.13188 (PMC10667648; doi:10.1111/1758-2229.13188)
Supplement: Supplementary file 1 — Table S1. Statistics of sequencing data. Table S2. Topological parameters of co‐occurrence networks of prokaryote communities. Figure S1. Pictures of sampling sites. Figure S2. Statistics of taxonomy annotation. Figure S3. Rarefaction curves. Figure S4. Species accumulation curve. Figure S5. Dominant prokaryotic phyla. Figure S6. Differences in dominant prokaryotic phyla among samples with different salinity. Different lowercases letters above each box in the same sub‐figure represent significant differences among samples from different salinity (Tukey's HSD test, p < 0.05). Figure S7. Relative abundances of dominant prokaryotic genera. Figure S8. Differences in dominant prokaryotic genera among samples with different salinity. Different lowercases letters above each box in the same sub‐figure represent significant differences among samples from different salinity (Tukey's HSD test, p < 0.05). Figure S9. Ratio of deterministic and stochastic processes to assembly of prokaryotic communities. [file EMI4-15-545-s001.docx]

#### **Supporting information for**

#### **Succession and assembly mechanisms of seawater prokaryotic communities along an extremely wide salinity gradient**

Xiaoyan Guan^#,1^, Zelong Zhao^#,1^, Jingwei Jiang^1^, Lei Fu^2^, Jiaojiao Liu^2^, Yongjia Pan^1^, Shan Gao^1^, Bai Wang^1^, Zhong Chen^1^, Xuda Wang^1^, Hongjuan Sun^1^, Bing Jiang^1^, Ying Dong^1^, Zunchun Zhou*^,1^

^1^ Liaoning Key Laboratory of Marine Fishery Molecular Biology, Liaoning Key Lab of Germplasm Improvement and Fine Seed Breeding of Marine Aquatic Animals, Liaoning Ocean and Fisheries Science Research Institute, Dalian, Liaoning 116023, PR China

^2^ Dalian Salt Chemical Group Co., Ltd, Dalian, Liaoning 116025, PR China

# These authors have contributed equally to this work.

* Corresponding information:

E-mail: [zunchunz@hotmail.com](mailto:zunchunz@hotmail.com) (Z. Z).

**Table S1.** Statistics of sequencing data.

| **Samples** | **Sequences** | **Bases (bp)** | **Average Length (bp)** |
| --- | --- | --- | --- |
| S3-1 | 35,664 | 14,709,429 | 412.44 |
| S3-2 | 34,837 | 14,434,468 | 414.34 |
| S3-3 | 39,799 | 16,462,918 | 413.65 |
| S3-4 | 35,129 | 14,502,739 | 412.84 |
| S3-5 | 39,258 | 16,203,502 | 412.74 |
| S3-6 | 38,193 | 15,789,628 | 413.41 |
| S6-1 | 36,346 | 15,059,420 | 414.33 |
| S6-2 | 35,724 | 14,856,060 | 415.85 |
| S6-3 | 33,594 | 13,951,507 | 415.29 |
| S6-4 | 39,346 | 16,296,996 | 414.19 |
| S6-5 | 39,747 | 16,522,226 | 415.68 |
| S6-6 | 36,689 | 15,170,572 | 413.49 |
| S9-1 | 38,298 | 15,762,957 | 411.58 |
| S9-2 | 35,273 | 14,469,653 | 410.21 |
| S9-3 | 36,890 | 15,271,690 | 413.97 |
| S9-4 | 41,626 | 17,079,347 | 410.3 |
| S9-5 | 37,293 | 15,293,013 | 410.07 |
| S9-6 | 33,741 | 13,767,151 | 408.02 |
| S11-1 | 35,846 | 14,567,797 | 406.39 |
| S11-2 | 38,185 | 15,560,475 | 407.5 |
| S11-3 | 34,255 | 14,153,770 | 413.18 |
| S11-4 | 34,891 | 14,208,902 | 407.23 |
| S11-5 | 41,173 | 16,787,301 | 407.72 |
| S11-6 | 34,801 | 14,187,441 | 407.67 |
| S13-1 | 36,867 | 14,860,676 | 403.08 |
| S13-2 | 38,615 | 15,576,031 | 403.36 |
| S13-3 | 34,112 | 14,018,475 | 410.95 |
| S13-4 | 35,095 | 14,112,234 | 402.11 |
| S13-5 | 38,750 | 15,606,181 | 402.74 |
| S13-6 | 33,831 | 13,723,808 | 405.65 |
| S15-1 | 40,741 | 16,705,131 | 410.03 |
| S15-2 | 33,596 | 13,772,608 | 409.94 |
| S15-3 | 39,799 | 16,490,142 | 414.33 |
| S15-4 | 39,510 | 16,199,055 | 409.99 |
| S15-5 | 36,727 | 15,104,565 | 411.26 |
| S15-6 | 36,742 | 14,918,746 | 406.04 |
| S17-1 | 37,769 | 15,081,794 | 399.31 |
| S17-2 | 41,004 | 16,336,894 | 398.42 |
| S17-3 | 41,307 | 16,763,389 | 405.82 |
| S17-4 | 35,583 | 14,177,610 | 398.43 |
| S17-5 | 38,814 | 15,485,428 | 398.96 |
| S17-6 | 40,369 | 16,139,691 | 399.8 |
| S19-1 | 38,152 | 15,303,599 | 401.12 |
| S19-2 | 33,996 | 13,697,942 | 402.92 |
| S19-3 | 34,400 | 14,091,559 | 409.63 |
| S19-4 | 41,740 | 16,716,793 | 400.49 |
| S19-5 | 41,166 | 16,494,372 | 400.67 |
| S19-6 | 39,407 | 15,757,692 | 399.87 |
| S21-1 | 33,757 | 13,567,869 | 401.92 |
| S21-2 | 40,541 | 16,437,253 | 405.44 |
| S21-3 | 35,870 | 14,444,287 | 402.68 |
| S21-4 | 38,123 | 15,279,585 | 400.79 |
| S21-5 | 35,005 | 14,070,175 | 401.94 |
| S21-6 | 37,860 | 15,082,873 | 398.38 |
| S23-1 | 33,571 | 13,423,279 | 399.84 |
| S23-2 | 41,581 | 16,761,411 | 403.1 |
| S23-3 | 39,402 | 15,736,516 | 399.38 |
| S23-4 | 39,812 | 15,853,690 | 398.21 |
| S23-5 | 38,433 | 15,376,792 | 400.09 |
| S23-6 | 41,229 | 16,299,668 | 395.34 |
| S25-1 | 35,240 | 13,962,224 | 396.2 |
| S25-2 | 35,827 | 14,322,684 | 399.77 |
| S25-3 | 35,609 | 14,076,476 | 395.3 |
| S25-4 | 33,295 | 13,226,691 | 397.25 |
| S25-5 | 33,754 | 13,331,520 | 394.96 |
| S25-6 | 37,151 | 14,656,799 | 394.51 |

**Table S2.** Topological parameters of co-occurrence networks of prokaryote communities.

|  | Empirical network | | | | | | | | | Random network | | | |
| --- | --- | --- | --- | --- | --- | --- | --- | --- | --- | --- | --- | --- | --- |
|  | Noses | Edges | Modularity | Average degree | Diameter | Density | Average path length | Clustering coefficient | Power-law model | Modularity (SD) | Average path length (SD) | Clustering coefficient (SD) | Small world coefficient  (SD) |
| S <16 | 45 | 351 | 0.300 | 15.600 | 3 | 0.355 | 1.699 | 0.737 | 0.897 | 0.156  (0.204) | 1.808  (0.014) | 0.339  (0.018) | 2.322  (0.106) |
| S >16 | 81 | 803 | 0.238 | 19.827 | 5 | 0.248 | 2.134 | 0.660 | 0.847 | 0.111  (0.180) | 2.006  (0.014) | 0.344  (0.013) | 1.807  (0.056) |

**Fig. S1.** Pictures of sampling sites.


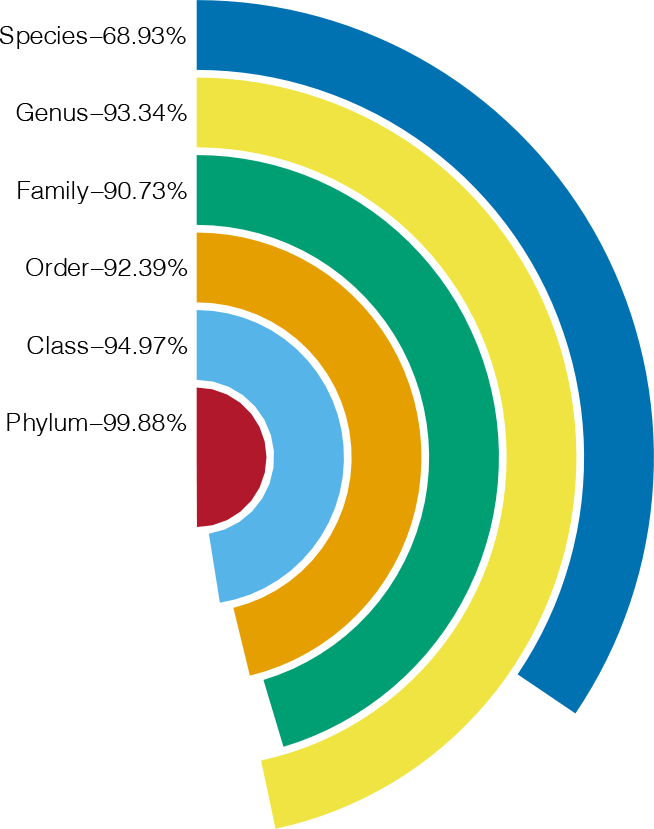


**Fig. S2.** Statistics of taxonomy annotation.


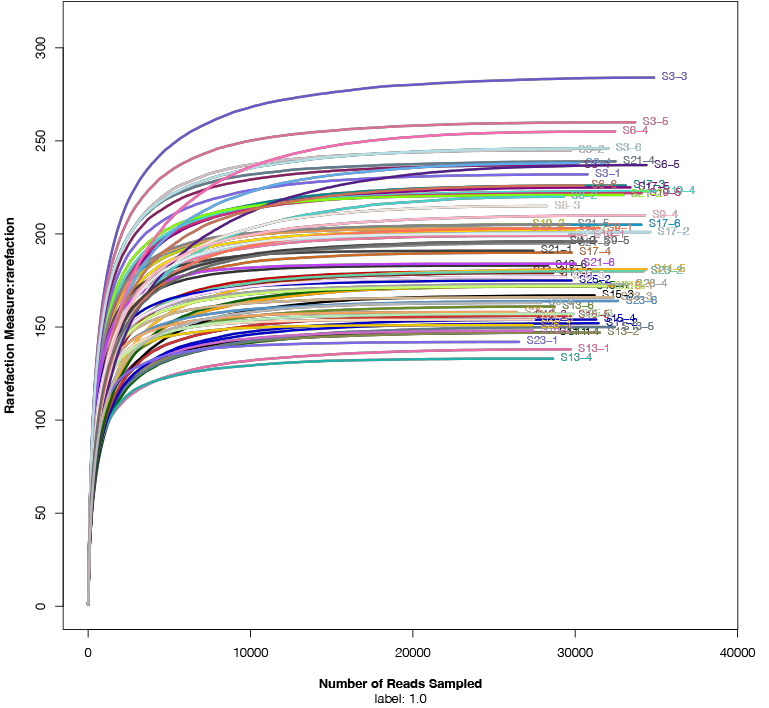


**Fig. S3.** Rarefaction curves.


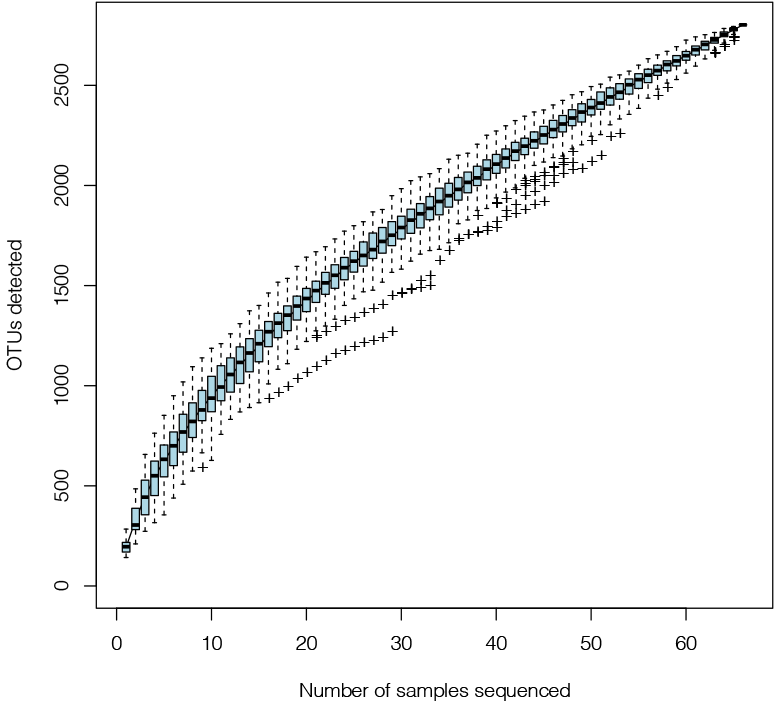


**Fig. S4.** Species accumulation curve.


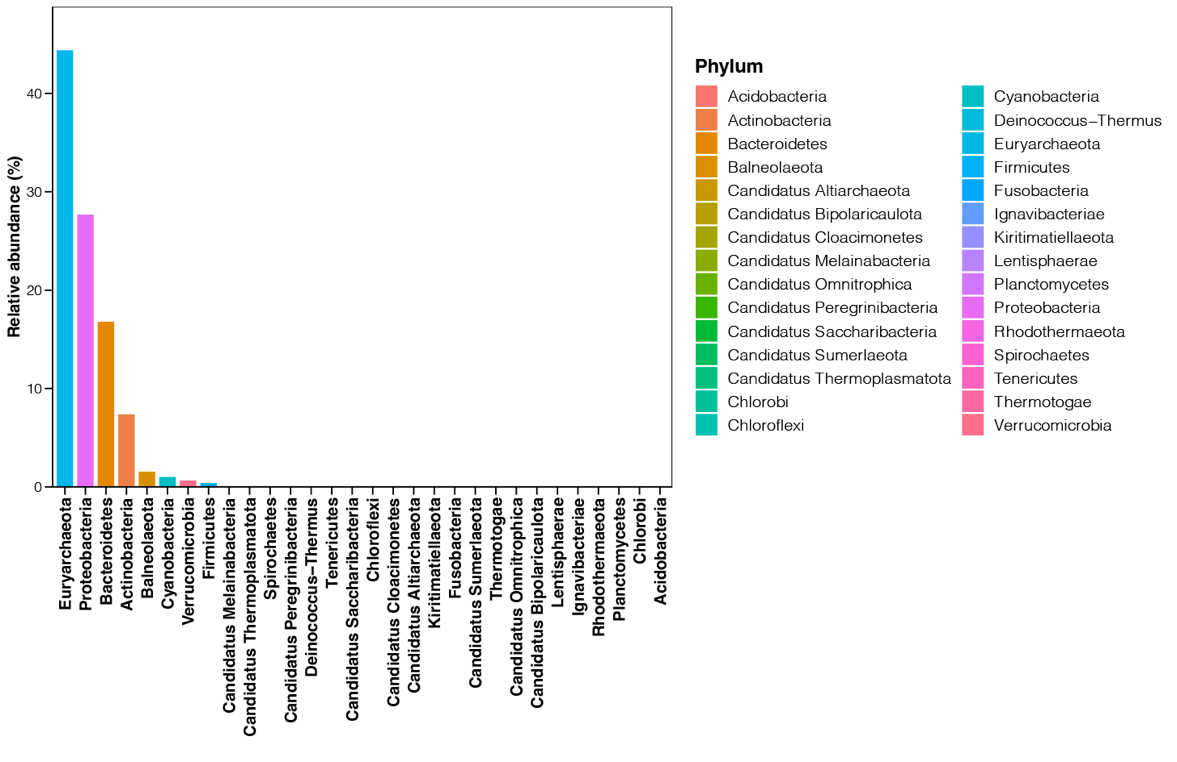


**Fig. S5.** Dominant prokaryotic phyla.


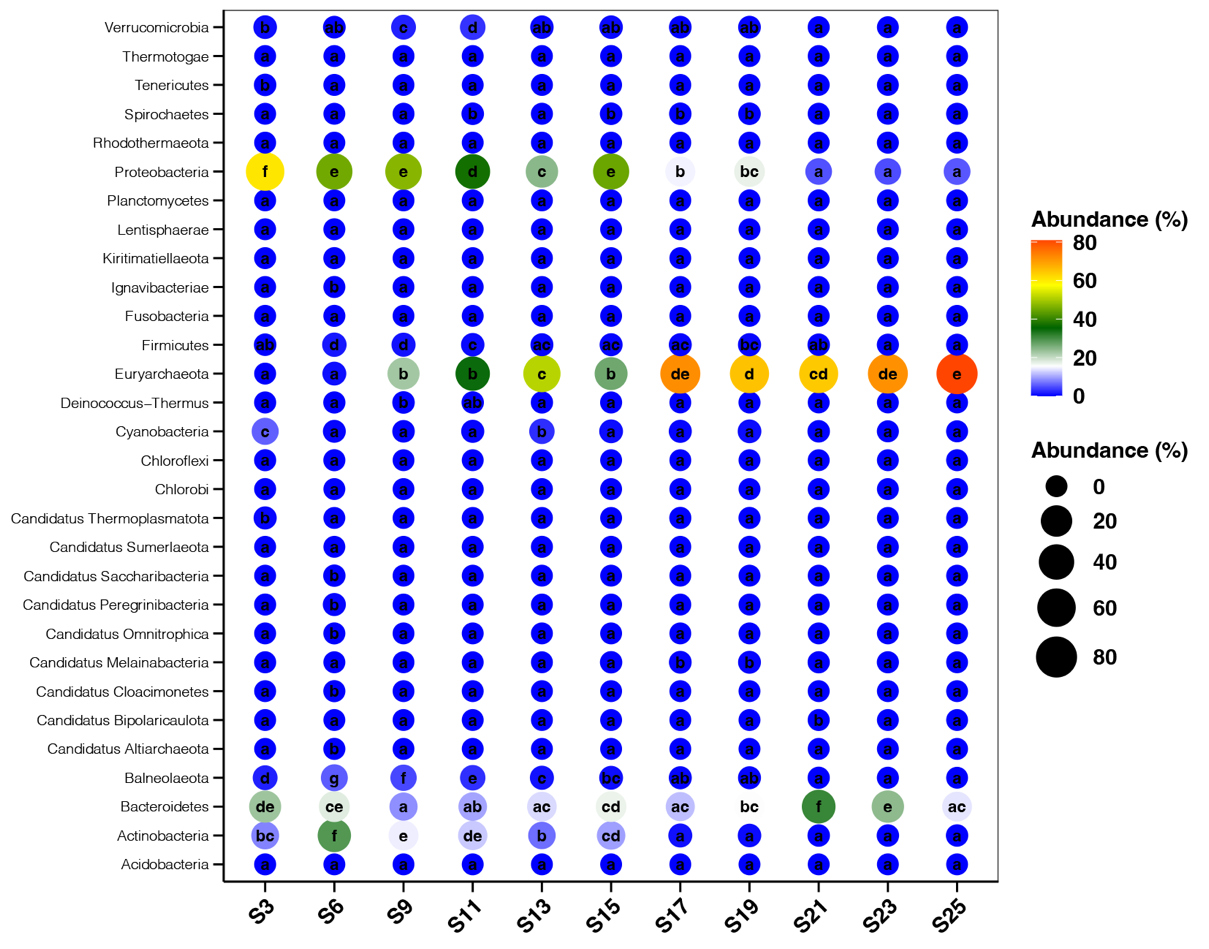


**Fig. S6.** Differences in dominant prokaryotic phyla among samples with different salinity. Different lowercases letters above each box in the same sub-figure represent significant differences among samples from different salinity (Tukey's HSD test, *p* < 0.05).


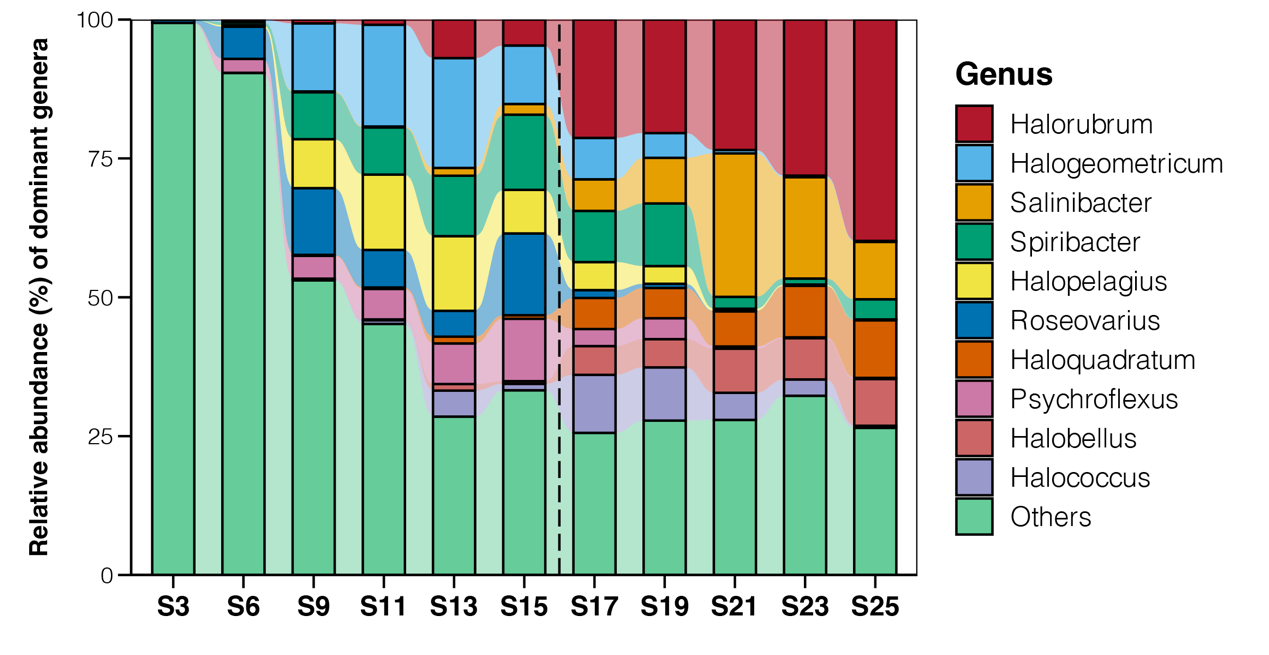


**Fig. S7.** Relative abundances of dominant prokaryotic genera.


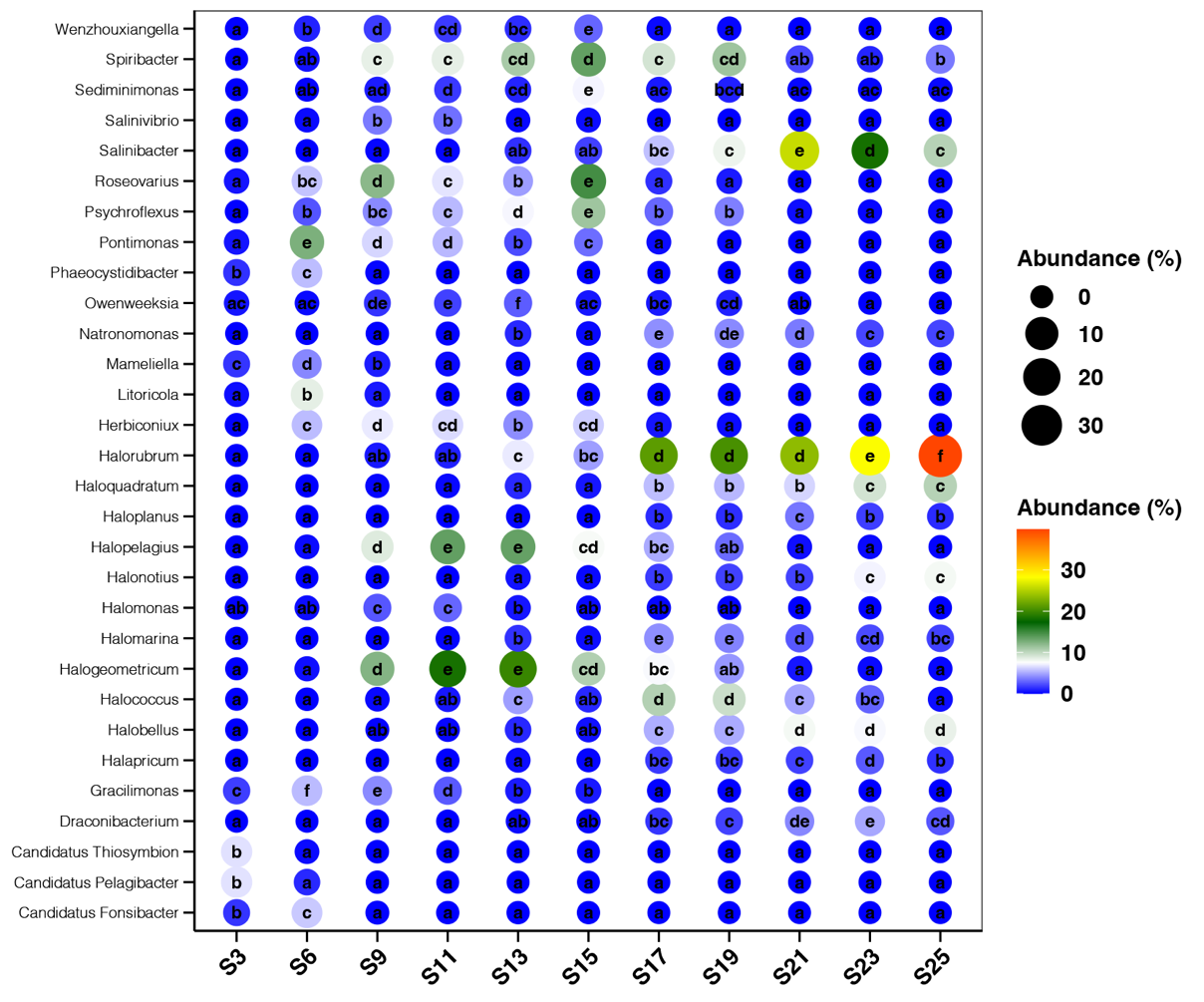


**Fig. S8.** Differences in dominant prokaryotic genera among samples with different salinity. Different lowercases letters above each box in the same sub-figure represent significant differences among samples from different salinity (Tukey's HSD test, *p* < 0.05).


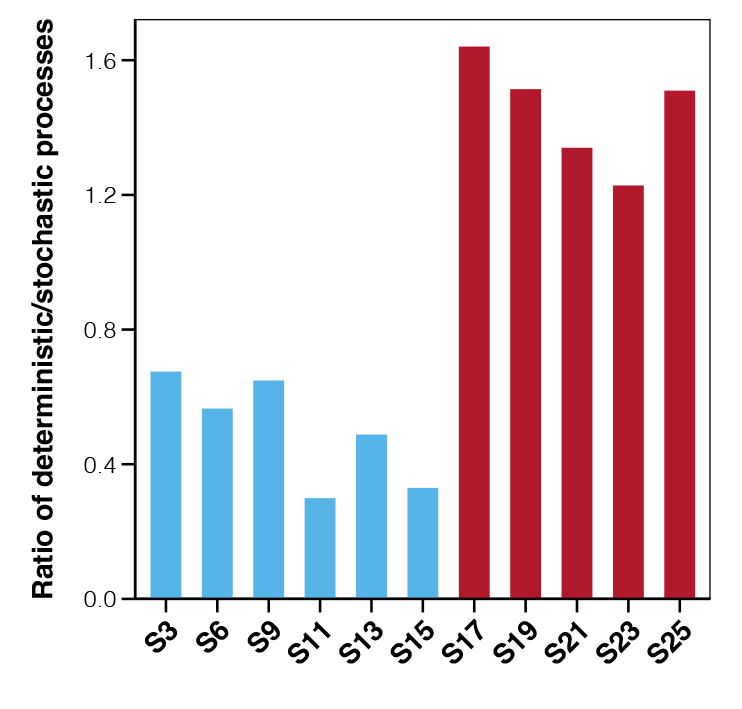


**Fig. S9.** Ratio of deterministic and stochastic processes to assembly of prokaryotic communities.
